# Brazilian natural gas market dynamics: A data panel analysis of the Brazilian market compared with Argentina, Colombia, Mexico, and India using Porter's five forces framework

**DOI:** 10.1016/j.heliyon.2024.e39190

**Published:** 2024-10-28

**Authors:** Hirdan Katarina de Medeiros Costa, Rafael Sacco, Clarissa Emanuela Leão Lima, Rodrigo Botão, Ciro Galvão, Gabriela Pantoja Passos, Thiago Brito, Giancarlo Ciola, Marcos Eduardo Melo dos Santos, Jewellord Nem Singh, Edmilson Moutinho dos Santos

**Affiliations:** a Institute of Energy and Environment, University of São Paulo, Brazil; b Department of Energy Engineering and Electrical Automation, Polytechnic School, University of São Paulo, Brazil; c International Institute for Applied System Analysis, IIASA, Austria; d Wilson Center, USA, and Institute of Asian Studies, Erasmus University of Rotterdam, the Netherlands

**Keywords:** Strategic evaluation, Market competition, Regulatory environment, Infrastructure investment, Economic development

## Abstract

This study offers a comparative analysis of natural gas markets in Brazil, Mexico, Colombia, Argentina, and India, spanning from 2000 to 2020. Leveraging panel data and historical time series analysis, coupled with Porter's Five Forces framework, it examines industry dynamics across upstream, downstream, midstream, and upstream dimensions. Insights into competitive landscapes are derived, considering regulatory frameworks, resource availability, infrastructure development, and demand patterns. Despite Brazil's efforts to enhance domestic production, infrastructure, and regulatory reforms, challenges persist, hindering sectoral growth. Comparative studies underscore the need for policy improvements to align with investment commitments. This study contributes novel insights into the competitive positioning of natural gas markets, offering actionable recommendations for stakeholders. Identifying potential bottlenecks informs strict decision-making in the global energy landscape.

## 1 Introduction

The global energy crisis, intensified by the post-pandemic recovery and the conflict in Ukraine, has driven up natural gas and oil prices, highlighting the importance of renewable energy and investments in energy efficiency and transition. Buildings with passive and hybrid systems, such as solar panels and wind turbines, as well as carbon capture systems for natural gas usage, are increasingly being implemented in new projects, particularly those related to electrification and green hydrogen ([[1], [2], [3], [4], [5]]).

Natural gas is considered the transition fuel for a more renewable and sustainable energy matrix for different reasons, such as gas-fired power plants, which can provide flexibility to the electrical energy system and ensure energy security by balancing the intermittency of renewable power generation sources. On the other hand, the relatively low cost of adapting industrial facilities that use more polluting energy sources, such as fuel oil and diesel, is another advantage for Energy Transition. Hence, the use of gas allows the reduction of greenhouse gas emissions when substituting more polluting fuels, which will play an essential role in meeting environmental commitments ([[6], [7], [8], [9]]).

This article aims to compare the performance of the natural gas market in Brazil with that of Mexico, Colombia, Argentina, and India. In addition to employing panel data and historical time series analysis from 2000 to 2020, the article establishes an analysis based on Porter's framework to scrutinise this industry across upstream, downstream, midstream, and upstream dimensions. Throughout this paper, we will delve into these aspects, offering comprehensive analyses and insights into the dynamics of the natural gas markets across these countries.

Numerous studies have explored natural gas demand and consumption across different geographic scales, with some focusing on emission predictions and investment trends ([[10], [11], [12]]). Evaluations of public policies related to natural gas have also been conducted, considering economic, environmental, or productivity perspectives ([13,14]). However, most of these studies overlook thermoelectric production. Only a few studies have focused on assessing the electricity industry from a productivity standpoint, with some examining infrastructure development ([15]) and others conducting energy system qualitative analyses. While these studies offer valuable insights, they do not provide federal government policies comprehensive analysis or compare different markets. Thus, our study fills this gap by providing a novel perspective.

Natural gas markets across these countries exhibit varying dynamics influenced by regulatory frameworks, resource availability, infrastructure development, and demand patterns. Brazil, for instance, has witnessed significant shifts in its natural gas sector, characterised by initiatives to enhance domestic production, infrastructure expansion, and regulatory reforms to foster competition ([16]). Mexico, Argentina, Colombia, and India have undergone their transformation, marked by liberalisation efforts in its energy sector, attracting foreign investment, promoting competition, developing infrastructure, and increasing market integration with ambitious renewable energy targets ([17]). By applying Porter’s Five Forces framework, the analysis delves into the competitive dynamics within each segment of the natural gas industry, considering factors such as the bargaining power of suppliers, the threat of new entrants, the bargaining power of buyers, the threat of substitutes, and competitive rivalry.

The development of natural gas industry's development difficulties in Brazil and these bottlenecks are more exhaustive than their production ([18]). Comparative studies have shown that this industry presents one of the best-expected investment portfolios. However, the sector's development expectations have yet to be met recently. The country has a level of consumption and production that is different from the potential of a country with gas reserves. Legislators have been committed to improving the legal framework to implement the announced investments.

There is a significant gap in comparative studies among developing countries, which needs to pay more attention to essential aspects when these countries are generically compared with developed countries.

This comprehensive approach allows for a nuanced understanding of the competitive landscape and strategic positioning of key players within the natural gas markets of the four largest Latin American Countries and India, thereby facilitating insightful comparisons and actionable insights for stakeholders in the global energy arena. Comparing Brazilian data with other countries with similar GDP PPP per capita can help identify whether the policies implemented in the country followed or diverged from the trend of similar countries ([19];^20^; [20]).

The objectives of this study are to evaluate Brazilian policies through data panel analysis and to conduct a strategy assessment using historical series analysis. A comparison with similar countries will identify whether the factors considered are exogenous or endogenous. Additionally, the analysis of twenty years' worth of historical data enables an assessment of policies applied in the medium and long term. A preliminary literature review (14,16,17) did not yield analyses from this perspective of the Brazilian gas market compared to other developing countries, indicating the novelty of this paper and highlighting a favourable gap for further research.

The article seeks to answer the following research question: What are the relationships between the transformations in favour of the competitiveness of the legal framework from the upstream to the downstream sectors of the Brazilian gas market?

This study constitutes an innovative contribution to the literature, demonstrating the industry's relative position in the general energy market. By broadening the understanding of the natural gas market in Brazil, the ability and likelihood of this sector to increase its share in the country's energy matrix become cleaner, as well as potential institutional, political or regulatory bottlenecks that might be out of sight of policy and decision-makers at this moment. This paper has the novelty of examining the recent shifts in Brazil's natural gas sector compared with other developing countries. It assesses their potential impacts on the upstream, midstream, and downstream segments, considering the domestic industry's competitiveness. The research outlines Porter's Five Forces methodology to analyse the strengths and obstacles within the Brazilian context, encompassing economic, logistical, and regulatory aspects. The quantitative and consolidated data for the analysed historical series are primarily focused on the years between 2000 and 2022. We used only specific data points from the year 2023.

The analysis in this paper reveals significant transformations in Brazil's gas industry over the past two decades. Brazil has transitioned from one of the most minor players to one of the most productive in gas generation and capacity compared to Argentina, Colombia, Mexico, and India. Despite increased electricity generation and oil production, the share of fossil fuels in the electricity grid has remained the same. Once monopolised by the State, upstream production gradually shifted, with Petrobras retaining dominance in gas generation and thermal power. However, challenges persist, including inadequate midstream infrastructure and disparities in regional consumption. Enhancing the regulatory framework, infrastructure development, market competition, and promoting the energy transition is imperative to address these challenges. By implementing targeted policies in these areas, Brazil can bolster the competitiveness and profitability of its natural gas industry while advancing its energy security and sustainability goals.

Following the literature review (section 2), methods (section 3) and discussion (section 4), results (section 5) applied Porter’s Five Forces Model to the analysis of the natural gas industry in Brazil: Bargaining Power of Suppliers (5.1), Bargaining power of customers (5.2), Threat of New Entrants (5.3), Threat of Substitution (5.4), and Degree of rivalry (5.5). The conclusion and policy recommendations are in the end (section 6).

## 2 Literature review

### 2.1 Theoretical background: the Porter’s five forces

**Fig. 1:**
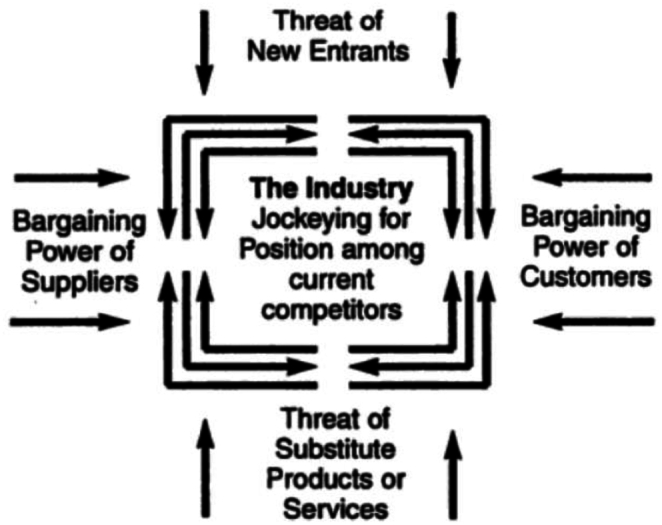
The five forces that shape industry competition (22).

In 1979, Michael Porter published his first article in the Harvard Business Review called “How Competitive Forces Shape Strategy”, where he explored the nature and degree of competition in an industry. According to Porter ([21]), knowing the sources of competitive pressure from different aspects of an industry can provide the baseline for a "strategic agenda of action". These aspects go beyond conventional industry rivals, being composed of five forces that shape industry competition: (i) suppliers' bargaining power, (ii) buyers' bargaining power, (iii) new entrant threat, (iv) substitute products or services threat, and (v) rivalry among existing competitors (Fig. 1) ([22]). These five forces define an industry's structure, and their strength determines its ultimate profit potential. A set of economic and technical features determines the strength of each competitive force.

The **bargaining power of suppliers** refers to the ability suppliers have to suppliers' ability value by charging higher prices, limiting quality or services, or shifting costs to other industry participants, determining its profit potential. A supplier group is considered robust if it is more concentrated than the industry it sells, serves many different sectors, offers differentiated or patented products, or provides a product with no substitute.

On the other hand, the **bargaining power of customers** reflects how much they can capture more value by imposing down prices or demanding better quality products or services. A group of customers is considered powerful when it is more concentrated than the suppliers from whom it buys, when a buyer purchases in large volumes concerning the size of a single vendor, particularly when the industry has high fixed costs, or when its products have no differentiation.

The first force is the **threat of new entrants** to an industry**.** New entrants bring new capacity to the market and seek to gain market share, putting pressure on prices and the rate of investments. This issue occurs because when the threat is high, incumbents hold down their prices or increase their investments to discourage new competitors, limiting the potential profit potential of an entire industry. This threat depends on how high the entry barriers are and on how timely the incumbents can react to new entrants.

The next force, the **threat of substitutes**, is observed when a substitute product serves the same demand and performs a similar function as an industry’s conventional product. The existence of substitute products limits an industry’s profit and growth potential. The sector must distance itself from them through product performance and marketing to avoid these market limitations. The threat of substitutes is higher when the buyer’s cost of switching to the substitute is low. It is essential to evaluate others' industry changes and development to be alert when their products can become attractive substitutes to consumers ([21]).

The fifth and last force is the **rivalry among existing competitors** in an industry. Such rivalry can be translated into price discounting, new product introductions, advertising campaigns, and service improvements. All those actions limit the industry's profitability potential (22). The level of rivalry in an industry is higher when competitors are numerous, industry growth is low (increasing the fights for market share), rivals' products are nearly identical, or when there is a combination of high fixed costs and low marginal costs, for example.

In addition, according to Porter ([22]), it is crucial to avoid “the common pitfall of mistaking certain visible attributes of an industry for its underlying structure.” The industry structure determines how the economic value is created and divided by the five forces mentioned above. In this sense, attributes such as governmental policies and complementary products or services are not forces since they are not bad (or good) for industry profitability. Both, for example, affect profitability through the way they influence the five forces.

Porter’s Five Forces Framework has already been applied to evaluate the industry’s structure and competitiveness in the energy sector. Yunna and Yisheng ([23]) used Porter’s Five Forces original model to assess the competitive landscape of the shale gas market in China. They analysed its variation tendency by setting different industry scenarios. The authors found that competition would become fierce after the Chinese government’s permission for private companies' activities in the national shale gas industry. In addition, shale gas has a substitution effect on coal and oil, and the rapid development of nuclear power in China represents a substitution threat to shale gas.

Hafezi et al. ([24]) applied Porter’s five forces to provide insights into the competitive global natural gas market landscape. They sought to identify the relevant forces that influence it, evaluating the competitive position of the National Iranian Gas Company (NIGC) competitive position. The authors highlighted the rapid market change in recent years and the better access to potential international markets offered by the interconnections associated with the modern LNG sector. Hafezi et al. (25) quantified the high level of competition faced by the NIGC and recommended developing robust and sustainable strategies to expand its market shares.

Aguilera and Inchauspe ([25]) used Porter's five forces approach to identify the economic market forces that shape the development of hydrogen as an energy carrier, discussing critical obstacles in the supply chain. The authors found that the distribution network is the significant fixed-investment barrier to market entry in the energetic hydrogen industry. However, this scenario could be overcome if natural gas infrastructure and technology are shared with the hydrogen sector.

In this paper, we propose using Porter's five forces to evaluate Brazil's natural gas market structure and assess its current scenario of competitiveness and profitability. The methodology is based on a qualitative analysis of the Brazilian natural gas market by 2022 and considering Porter's Five Forces: (i) bargaining power of suppliers; (ii) bargaining power of customers; (iii) threat of new entrants; (iv) threat of substitutes; (v) rivalry among existing competitors.

### 2.2 Oil and gas industry in Brazil

The literature on energy policy in Brazil highlights federal reforms since the 1990s, with recent studies focusing on energy security and corruption following the discovery of pre-salt oil ([[26], [27], [28], [29], [30]]). Comparisons with Norway and the UK are drawn due to similar geological characteristics (31).

The shift from a Petrobras monopoly to a competitive market began during the Cardoso government (1995–2003), with gradual liberalisation continuing through subsequent administrations (27). The discovery of offshore reserves in 2007 did not impede market liberalisation (31). Factors driving this trend include high oil prices and changes in the government's perception of risk (29).

Legislation such as the Pre-Salt Law aimed to increase state control, though debates persist on state-led development versus private investment (28). Petrobras faced challenges, including forced fuel price reductions and debt pressures, leading to divestments ([31]). Rousseff's presidency saw economic recession and a corruption scandal, culminating in her impeachment in 2016 ([26,32]).

Temer's administration accelerated energy market liberalisation, encouraging private investment and reducing regulatory burdens ([27]). Brazil saw a resurgence in oil and gas exploration during this time, with major international companies participating ([33]).

The downstream segment also saw changes, with initiatives to promote competition and attract investments ([34]). Despite reduced Petrobras participation, it maintains a significant role in exploration (27).

Concerns persist over fuel imports and crude oil exports' impact on development 38). Record investments in pre-salt fields and improvements in bidding processes indicate Brazil's attractiveness to investors (34).

Initiatives to reduce regulatory burdens aim to attract foreign and national capital, although decreased refinery utilization rates and increased fuel imports pose challenges (32). Supporting smaller Brazilian firms could mitigate these challenges, but refinery disinvestment may hinder their growth ([35,36]).

## 3 Methods

### 3.1 Data panel

The correlation between GDP per capita and energy consumption is frequently observed, with economic advancement often paralleled by heightened energy usage. Traditional economic growth entails augmented industrial output, infrastructure enhancement, and elevated living standards, all necessitating sustained energy provision. As posited by Kuznets' Law ([37]), energy consumption tends to surge as a nation progresses economically, peaking during intermediate developmental stages before tapering off as the economy achieves greater efficiency and diversification.

Our research contribution centred on the examination of Brazil's natural gas industry. The selection criteria for these countries encompass the largest economies in Latin America (Brazil, Argentina, Mexico, and Colombia), supplemented by India due to its nominal GDP and PPP, nominal per capita GDP and PPP, as well as recent industrialisation, hydrocarbon production, and GDP PPP (including per capita), akin to those of Latin America (17).

Employing a mixed methodology, this study constitutes a case analysis. The qualitative application of Porter's Five Forces and the examining the legal framework transformations form the crux of this paper. The reforms instituted within Brazil's gas sector align closely with those observed in developed nations, primarily aiming for heightened competition. The deregulated market landscape engenders an increase in stakeholders and infrastructure investments, resulting in greater price competitiveness ([38,39]).

The theoretical insights are juxtaposed with quantitative data from international and Brazilian governmental and non-governmental energy agencies. Consistent with this paper, scholarly articles published in reputable journals have explored the Brazilian context, offering qualitative and quantitative contributions (16).

Data will be compared across five chosen countries based on absolute figures, per capita values, and participation indicators in the energy market. Moreover, global indicators will also be taken into account. Consequently, generating 14 indicators spanning 20 years (2000–2020) for 20 countries, in addition to the selected nations and global indicators, is feasible. These indicators include.

● Natural gas Electricity Installed Capacity (MW)
● Natural gas Electricity Generation (GWh)
● Natural gas proved reserves
● Gas production (MWh)
● Gas Consumption - TWh
● CO2 emissions (kt)
● Population
● Natural gas Electricity Installed Capacity (MW) per capita
● Natural gas Electricity Generation (GWh) per million resident
● Electricity from fossil fuels kWh per capita
● Natural gas proved reserves per capita
● Gas production (MWh per capita)
● Gas Consumption - MWh per resident
● marketed production of natural gas by country (million standard cu m)
● CO2 emissions (kt) per 1000 residents

Utilising per capita production data for renewable and non-renewable energy sources is a more apt approach when comparing the chosen nations. The strategic evaluation of public policies in Brazil within the framework proposed by Porter (22) should rely upon energy data indicators spanning various energy outlets in contrast to its counterparts in Latin America. By considering per capita indices, the absolute disparities in population, economy, territorial expanse, and production are mitigated, thus facilitating a more equitable comparison. The selection of countries with similar GDP per capita holds significance as it serves as a pivotal determinant for energy demand and the capacity for investment in emerging energy production and consumption paradigms, encompassing transitions towards innovative technologies like photovoltaic, wind, hydrogen, biofuels, and waste-to-energy systems (17).

Moreover, the inclusion of oil and gas reserve data assumes importance in the analytical process, as the accessibility of these resources significantly influences investments in renewable energy, owing to the ramifications of public policy dispersion. Subsequent research endeavours could incorporate variables such as oil prices and investment rates in renewable and non-renewable energy sectors. These variables, in turn, notably impact a nation's efficacy in fostering the evolution or transition of its energy portfolio towards cleaner alternatives.

The application of Porter's (22) five forces framework to comparatively analyse the gas market in Brazil, from downstream to upstream, concerning other developing countries, reveals crucial insights into the competitive dynamics and challenges market participants face. Downstream, the entry of new competitors is influenced by governmental regulation, infrastructure costs, and contractual agreements, while upstream, factors such as access to natural resources, technology, and investment in exploration play a crucial role. Price negotiations and contracts, the influence of major international players, and governmental policies of encouragement or restriction are determining aspects. Comparative analysis identifies competitive advantages and areas of vulnerability in each market, providing valuable insights for strategic decision-making and public policies aimed at the gas sector across different economic development contexts.

In terms of limitations, future developments of this research include creating a computational mathematical model to analyse the influence of the international oil barrel price and international interest rates on energy investments in the selected countries. Additionally, another limitation and further research development suggest the creation of a computational engine to assess the multiple internal influences among the indicators numerically. These engines facilitate the understanding of the impacts of public policies in the medium and long term.

## 4 Discussion: from state complete control to market liberalisation

The origin of the natural gas industry in Brazil mixed up with the creation of Petrobras in 1953. The state-owned company started controlling the entire value chain as part of a vertically integrated monopoly. In 1997, the Petrobras monopoly was broken, and a regulatory agency was created (ANP, the National Agency of Petroleum, Natural Gas and Biofuels, an autarchy under a special regime linked to the Ministry of Mines and Energy) and other companies started their operations in Brazil (18 In the case of natural gas, article 177 of the Federal Constitution specifies the upstream and midstream of the gas market as the Federal Union’s competence, while the downstream, specifically, the distribution of gas using pipelines, as the State's competence ([40]). Then, Law 9478 of 1997 regulated the gas industry at the federal level and Law 11,909 was enacted to regulate the gas midstream.

ANP became responsible for adopting mechanisms to stimulate the competitiveness and efficiency of the natural gas market, ensuring its proper functioning and avoiding anti-competitive practices that may constitute violations of the economic order ([18,[41], [42], [43]]).

Brazilian States have adopted different models for regulating local piped gas services and granting the service ([44]). The execution of the services was given, in most cases, to mixed capital companies, with the presence of the Granting Authority in the corporate bodies ([45]).

There needed to be more than the changes in the Legislation to secure new players in the industry and simultaneously create more competition. In practice, the control exercised by Petrobras over the natural gas industry and its value chain continued to discourage the entrance of new players. Unlocking the development and growth of the natural gas market requires less concentration and marketing opening ([46]).

Changes in the gas industry structure were required and would only be achieved with a clear strategy, government policies and influencing, developments in the regulatory framework and market unbundling ([[47], [48], [49]]). "Gás para Crescer" and "Novo Mercado do Gás" programs consolidate the federal government's response to trying to unlock the growth of the country's natural gas industry. Regulatory aspects diversify infrastructure and expand the number of agents, demanding well-oriented policies to encourage investment in the entire natural gas production chain. In a coordinated fashion, those programs were translated into new ANP regulations, guidelines from the National Energy Policy Council (CNPE), the Cessation Commitment Term (TCC), signed by Petrobras and CADE, New Gas Law (Law 14,134/2021), which updates the regulatory framework throughout the piped gas chain ([50]). As the New Gas Law consolidates all the liberalising efforts discussed over the last few years, it is expected that a new dynamic in the natural gas market will be reinforced, allowing multiple agents to act throughout its chain ([51,52]).

In 2019, to end the Petrobras monopoly in practice, the national oil company and the Brazilian Administrative Council for Economic Defence (CADE) signed a Cessation Commitment Term (TCC) through which Petrobras has committed itself to sell its shares in natural gas midstream and downstream companies, such as *Nova Transportadora do Sudeste* (NTS), *Transportadora Associada de Gás* (TAG), *Transportadora Brasileira Gasoduto Bolívia-Brazil* (TBG) and Gaspetro (48; 52). After the completion of the divestment procedure of Gaspetro by Petrobras in July 2022, the new owners assumed Compass (with 51 % of the shares) in conjunction with Mitsui Gás e Energia (with the remaining 49 %) ([53,54]). According to the CADE, these measures would give access to the infrastructure and market gas to other producers, who would no longer need to sell to Petrobras, encouraging the free market (48).

Federal Law No. 14,134/2021 and Decree No. 10,712/2021 established the main rules related to gas commercialisation and clarified what market aspects follow under Federal or State competence (Brazil, 2021a). At the federal level, ANP authorised commercialisation activity. Additionally, each State may have a regulatory body that authorises or registers gas traders (52).

Also, at the federal level, it is noteworthy that ANP Resolution No. 52/2011 regulated the authorisation for the commercialisation of natural gas within the sphere of competence of the Union. To this end, the requirements for obtaining ANP’s authorisation were determined for the commercialisation of fuel, in addition to conceptualising the figures of the self-producer, the self-importer and the free consumer ([42,53]).

In addition to the separation and clear distinction between the activities of piped gas distribution services and commercialisation of the molecule, it is necessary to assess whether there are and what are the rules defined in favour of establishing a competitive free commercialisation market for natural gas, an essential condition for the country to be able to boost its market and achieve economic and social benefits resulting from it ([[55], [56], [57]]).

## 5 Results: natural gas industry analysis in Brazil applying Porter's Five Forces Model

### 5.1 Bargaining power of suppliers

From the upstream perspective, Brazil's national natural gas production has increased in the last eight years, and many companies have participated in exploration and production activities. However, even with the decline of the Petrobras market concentration in concession criteria (70 % in 2021), the Brazilian national oil company still has a large share in the sector and a relatively stable market position in operations and processing capacity criteria (around 92 % in 2021) (Table 1, Table 2). Foreign multinational companies prefer to enter as concessionaires with Petrobras as the operator. On the other hand, Brazilian private national companies are small in size and are more focused on onshore operations.

**Table 1:** Natural gas production by concessionary and operator - 2021.

Ranking	Concessionary	Natural Gas (thousand m3)	Share	Ranking	Operator	Natural Gas (thousand m3)	Share
1	Petrobras	35.332.345,4	72,4 %	1	Petrobras	44762364,66	91,7 %
2	Shell Brasil	5.566.983,2	11,4 %	2	Eneva	2165197,331	4,4 %
3	Eneva	2.165.197,3	4,4 %	3	TotalEnergies EP	742016,6095	1,5 %
4	Petrogal Brasil	1.661.172,0	3,4 %	4	Origem Alagoas	200614,7047	0,4 %
5	Repsol Sinopec	1.112.387,5	2,3 %	5	SPE Miranga	134396,8165	0,3 %
6	TotalEnergies EP	640.206,9	1,3 %	6	Shell Brasil	117903,4338	0,2 %
7	Enauta Energia	556.014,7	1,1 %	7	Potiguar E&P	109559,835	0,2 %
8	Equinor Energy	359.022,5	0,7 %	8	Alvopetro	108308,6101	0,2 %
9	Petronas	169.905,5	0,3 %	9	Trident Energy	80966,5594	0,2 %
10	CNODC Brasil	151.797,0	0,3 %	10	Imetame	75274,1095	0,2 %
	Other 52 agents	1.109.295,0	2,3 %		Other 30 agents	327724,2945	0,7 %
	**Total**	**48.824.327,0**	100,0 %			**48.824.327,0**	100,0 %

**Table 2:** Natural gas processing capacity by producing poles, December 2023.

Processing units	Location (State)	Startup	Nominal capacity 103 m3/day	Control	Share
Urucu	Coari (AM)	1993	12.200	Petrobras	92.2 %
Lubnor	Fortaleza (CE)	1987	350		
Guamaré	Guamaré (RN)	1985	5.700		
Origem Energia	Pilar (AL)	2003	1.800		
Catu	Pojuca (BA)	2022	2.000		
Estação Vandemir Ferreira	São Francisco do Conde (BA)	2007	6.000		
Cacimbas	Linhares (ES)	2008	18.100		
Sul Capixaba	Anchieta (ES)	2010	2.500		
Reduc	Duque de Caxias (RJ)	1983	5.000		
Cabiúnas	Macaé (RJ)	1987	24.600		
Caraguatatuba	Caraguatatuba (SP)	2011	20.000		
Alvopetro	Mata de São João (BA)	2020	500		
Cubatão	Cubatão (SP)	1993	2.300		
Eneva	Santo Antônio dos Lopes (MA)	2013	8.500	Eneva	7.7 %
Caburé	Caburé (BA)	2020	0.500	Alvopetro	<1 %
Total			109.55		100 %

Table 3 shows several key trends in the Brazilian natural gas sector between 2014 and 2023. Imports have shown significant fluctuation over the years, with an evident decline from 17.40 to 6.47 units, suggesting a reduction in reliance on imported gas despite some notable increases during specific years. In contrast, natural gas reinjection has steadily increased from 5.74 units to 28.77 units, highlighting a growing focus on reinjection for secondary recovery in oil fields. When comparing onshore to offshore production, there is a marked difference. Onshore production has remained relatively stable, with slight fluctuations ranging from 7.4 to 8.7 units, indicating no significant growth.

**Table 3:** Brazilian natural gas balance – 2014–2023.

Natural Gas (billions m³)	2014	2015	2016	2017	2018	2019	2020	2021	2022	2023
Imports	17,40	19,11	13,32	10,64	10,84	9,86	7,87	16,90	8,98	6,47
Sales^1^	31,77	32,40	27,22	27,49	26,05	25,85	21,97	30,33	22,37	20,26
NGL^2^	1,51	1,38	1,54	1,85	1,90	1,96	1,50	1,38	1,41	1,40
Own consumption³	7,93	9,67	8,76	8,93	8,75	8,89	9,03	9,57	9,18	9,19
Gas flaring and losses	1,62	1,40	1,48	1,38	1,36	1,59	1,23	1,23	1,27	1,41
Reinjection	5,74	8,87	11,07	10,08	12,81	15,78	20,01	22,21	24,97	28,77
Natural gas production Onshore	8,5	8,3	8,7	7,8	8,0	8,2	7,4	8,2	7,4	7,7
Natural gas production Offshore	23,3	26,	29,1	32,2	32,8	36,4	39,1	40,5	42,9	46,8
Pre-salt	6,2	10,6	14,4	18,1	21,0	25,9	30,6	32,9	36,0	40,8
Post-salt	17,1	16,1	14,7	14,0	11,8	10,5	8,5	7,5	6,9	6,0
Onshore reserves	71,21	70,99	63,57	65,97	69,02	68,64	77,73	77,64	99,01	100,57
Offshore reserves	399,88	359,60	315,51	303,11	299,46	296,43	261,09	303,55	307,51	416,51

In contrast, offshore production has consistently increased from 23.3 units to 46.8 units, underscoring the growing importance of offshore fields, especially in deeper waters. Pre-salt fields have shown substantial growth within offshore production, increasing from 6.2 units to 40.8 units, highlighting their strategic priority due to their considerable, untapped potential and relatively recent development. Meanwhile, post-salt production is declining from 17.1 to 6.0 units, suggesting these fields are maturing or reaching the end of their productive life. Onshore reserves have varied but shown a promising increase in recent years, rising from 71.21 units to 100.57 units, likely due to enhanced recovery techniques or new discoveries. Offshore reserves have also followed a fluctuating but generally positive trend, with an increase from 261.09 units in 2016 to 416.51 units in the latest year, attributed to discoveries and technological advancements in offshore drilling.

In summary, the Brazilian natural gas sector is experiencing a transition characterized by declining import dependency, increased reinjection practices, significant growth in offshore and pre-salt production, and an overall rise in both onshore and offshore reserves. This trend indicates that Brazil is enhancing its self-sufficiency and capitalizing on its substantial offshore and pre-salt resources. The sales data, however, highlights the need for public policies to promote internal market sales despite the increase in production.

**Fig. 2:**
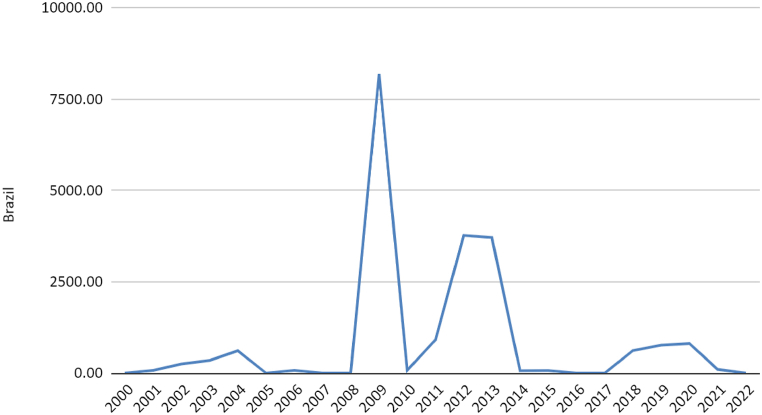
Public investments (2021 million USD) in natural Gas energy in Brazil by year. Source ([59]).

Another significant factor is that state investment in gas or electricity production using gas is nearly insignificant in India, Argentina, Mexico, and Colombia. The Brazilian government undoubtedly leads in sector investment among the selected countries. The market opening has partially excluded state investment, as occurred in the other compared countries. The expansion of Brazil's production and installed capacity by natural gas between 2000 and 2012 is directly related to these state investments via Petrobras (15) (Fig. 2).

When it comes to natural gas imports, Brazil imported 7.9 billion m³ of natural gas in 2020, of which 6.6 billion m³ (around 83.2 %) came from Bolivia through the Gasbol pipeline and the rest in the form of liquefied natural gas (LNG) from other countries, such as the United States (0.91 billion m³) and Trinidad and Tobago (0.16 billion m³) ([60,61]). Besides this significant dependence on Bolivian gas in 2018, 2019, and 2020, Bolivian gas imports in 2021 remained steady, around 20 million m³/day, while the importation volume from Argentina via pipeline and other countries via LNG has increased slightly, reducing the dependency and risk on one major supplier ([62,63]).

**Fig. 3:**
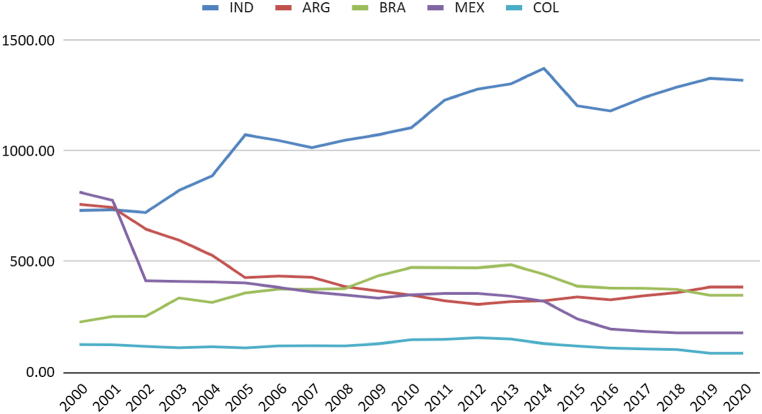
Natural gas consumption (thousand m3). Source: (59).

**Fig. 4:**
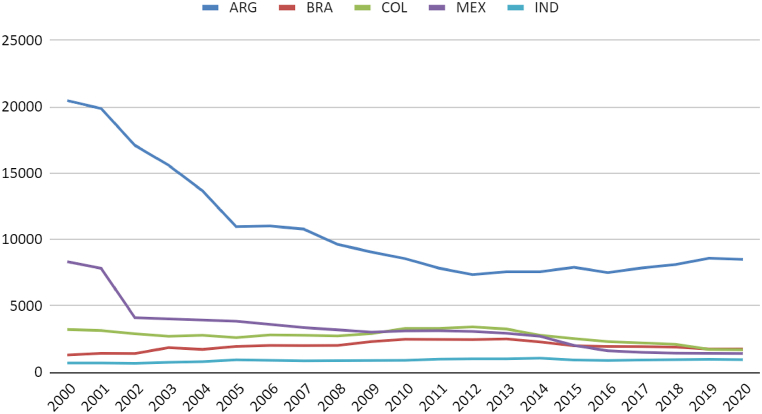
Natural gas proved reserves (thousand m3) per capita. Source ([64]).

**Fig. 5:**
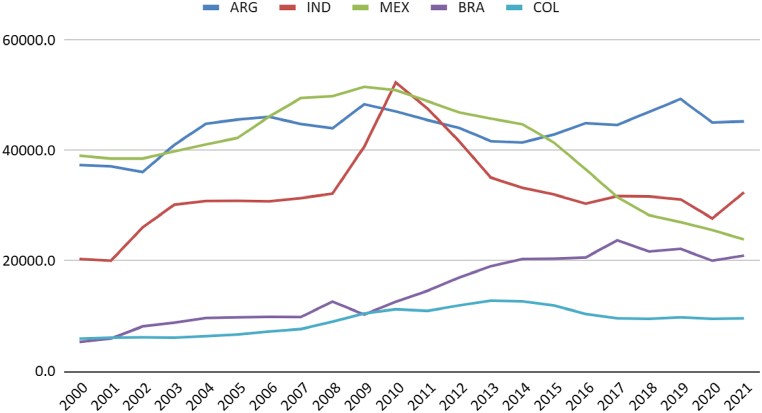
Marketed natural gas production by country (million standard cu m). Source: (59,60).

Brazil's total natural gas reserves are comparable to those in Argentina (Fig. 3). Although the per capita availability in the South American neighbour is higher (Fig. 4), it is noticeable that Colombia, Brazil, and Argentina have maintained production close to consumption levels. Mexico and India have increased consumption without possibly increasing production (Fig. 5). It is regrettable that, except for India, the countries analysed possess sufficient natural resources not to require imports yet still exhibit consumption levels surpassing production.

Although Petrobras is still a dominant supplier, the increasing presence of other participants is starting to show results, according to Fórum do Gás ([65]), in 2022, new suppliers began to offer gas supply agreements with prices up to 42 % lower than Petrobras’ ones. The low share of imported gas in Brazilian consumption can be traced back to an initiative from the early 2000s involving the construction of gas-fired power plants due to difficulties in electricity supply stemming from a drought that had impacted the hydroelectric sector. In fact, since the 2010s, Brazil has had a much greater gas supply than demand. Some of the gas is even reinjected into oil production fields to maintain productivity in mature fields.

**Fig. 6:**
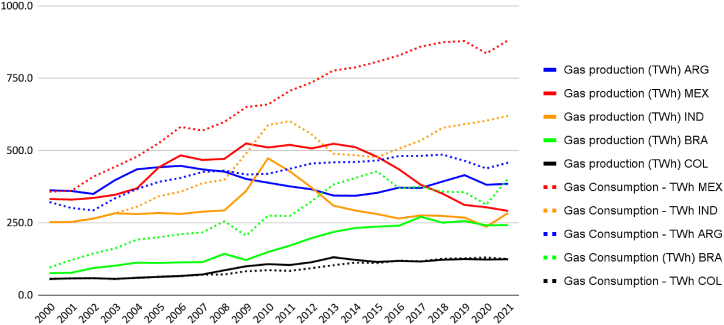
Gas production and Consumption (Twh). Source: (59,60).

**Fig. 7:**
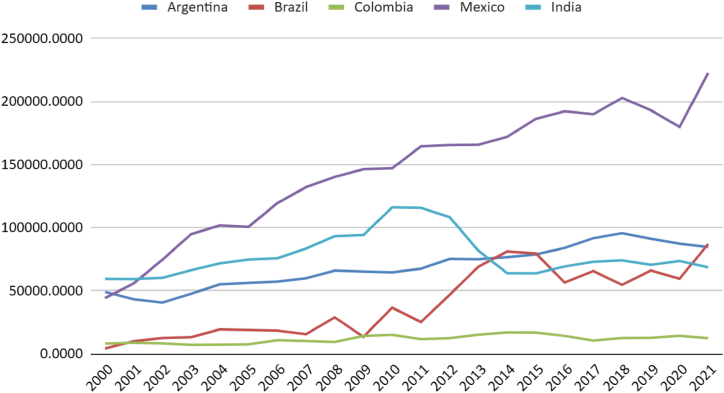
Natural gas electricity generation (GWh). Source: (59,60).

This distortion is evident when comparing the data of gas traded by Brazil with other countries (Fig. 5, Fig. 6, Fig. 7). Almost half of Brazil's gas is commercialised. Consequently, Brazil does not require gas imports for the interconnected pipeline network. Brazil, however, depends on the import of LNG (Table 4). However, regions still need to be connected by pipelines, and the inability to liquefy all gas domestically still renders the country dependent on imports. The gas supply in Brazil presents not only an opportunity for import substitution but also for exporting liquefied gas if there are projects aimed at increasing the capacity of gas distribution and refining (15).

**Table 4:** Brazilian Natural gas imports (106 m³).

**Countries**	Brazilian Natural gas imports (106 m³)									
	**2014**	**2015**	**2016**	**2017**	**2018**	**2019**	**2020**	**2021**	**2022**	**2023**
**Total (a)+(b)**	**17.398**	**19.112**	**13.321**	**10.643**	**10.842**	**9.855**	**7.874**	**16.974**	**8.985**	**6.469**
**Natural Gas (a)**	**12.049**	**11.854**	**10.369**	**8.886**	**8.071**	**6.795**	**6.551**	**7.391**	**6.384**	**5.628**
Argentina	67	169	–	–	–	–	–	68	–	–
Bolivia	11.981	11.684	10.369	8.886	8.071	6.795	6.551	7.324	6.384	5.628
**Liquified Natural Gas (LNG)**	**5.349**	**7.258**	**2.952**	**1.756**	**2.771**	**3.061**	**1.323**	**9.583**	**2.601**	**841**
Algeria	–	80	–	–	–	–	–	–	–	37
Angola	89	–	91	362	89	91	93	135	–	–
Argentina	–	–	–	–	–	–	130	–	–	–
Bahamas	–	–	–	–	–	82	–	–	–	–
Belgium	35	78	81	–	277	–	–	–	14	–
Cameroon	–	–	–	–	–	105	–	–	–	–
China	–	–	–	–	–	–	–	–	–	59
Equatorial Guinea	465	176	162	–	–	92	–	–	68	–
France	–	131	–	82	87	–	–	–	–	–
Netherlands	285	147	–	–	5	191	–	89	–	–
Nigeria	1.505	1.829	1.095	730	351	345	27	21	81	–
Norway	576	823	252	–	242	251	–	–	–	–
Portugal	221	250	–	–	–	–	–	–	–	–
Qatar	170	1.366	655	124	171	–	–	853	124	–
Singapore	–	–	–	–	–	–	–	–	18	–
Spain	455	372	–	–	–	–	–	–	52	57
Trinidad and Tobago	1.479	1.764	273	81	818	573	161	249	–	24
United Arab Emirates	–	62	–	–	–	–	–	14	–	–
United Kingdon	–	89	75	–	–	–	–	–	–	–
United States of America	71	92	266	376	730	1.331	912	8.222	2.244	663

Regarding midstream and downstream sectors, Petrobras owns 99,54 % (or 107.210.000 m³/day) of Brazil's natural gas processing capacity and 14 of the 15 Brazilian Natural Gas Processing Units (NGPU) in 2020. In the same year, the only private NGPU was operated by Alvopetro, located in the city of Mata de São João, State of Bahia – Northeast Brazil, and it was responsible for only 0,46 % (or 500.000 m³/day) of the natural gas processing capacity in the country ([60]).

Despite Federal Government efforts and ambition to develop a more competitive market for the natural gas industry and foster investments in offloading and transmission infrastructure of natural gas by other market agents than Petrobras, this scenario shows Brazil's concentrated natural gas processing industry and the vast Petrobras' bargain power as the primary supplier.

### 5.2 Bargaining power of customers

The Brazilian natural gas demand is formed mainly by (i) demand from local distribution companies (LCD), (ii) consumption of refineries and fertiliser plants (FAFEN, from the Portuguese "Fábrica de Fertilizantes"), and (iii) consumption of thermal power plants ([66]), being LCD is responsible for final consumer delivery in the country in general.

Concerning the electricity sector, due to droughts in the early 2000s, Brazil began the process of structuring thermal power plants and gas pipelines, including connecting to the Bolivian network, which was then one of the largest providers of natural gas in South America — with the development of natural gas production associated with oil in the ultra-deepwater pre-salt fields, thermal power plants started to be predominantly supplied by domestic gas. Thermal power plants exhibit variable production levels over the years and typically serve as an energy security factor during dry periods when Brazil relies heavily on hydroelectric production (15). The peak use of hydroelectric plants was observed from 2014 to 2016 due to severe droughts (Fig. 9, Fig. 11). From 2019 onwards, with a significant increase in wind and solar sources, thermal power plants have operated only as support for the national integrated system when renewable electricity production is lower. Additionally, electricity consumption is for industrial purposes, based on thermal power plants near Brazil's ports and coastal gas pipelines. Until 2012, Petrobras constructed thermal power plants as part of the process of increasing installed capacity. However, starting from the political-economic crisis during the second term of Dilma's government, the state-owned company began selling thermal power plants to the private sector. The increase in installed capacity became predominantly the private sector's responsibility, particularly during the Temer administration (16).

When comparing the Brazilian case to other countries, a deceleration in the growth rate of installed capacity is observed in Argentina, moving from levels similar to India and Mexico to levels similar to Brazil. Brazil, in turn, has moved from performance similar to Colombia to approach the Argentine level. Colombia has stagnated its natural gas electricity production over the last 20 years (Fig. 6).

Regarding generation, it is noted that the Mexican system generates nearly double the electricity compared to Argentina, Brazil, and India. Colombia even shows a reduction in generation. In the case of Brazil, less consistent performance is observed compared to others due to the complementary function of the thermoelectric system for renewable sources, generating more energy in drier periods of the historical series (Fig. 7).

**Fig. 8:**
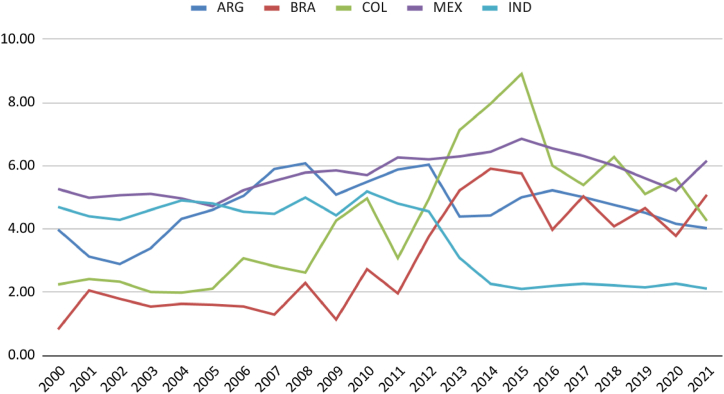
Natural gas Electricity Installed Capacity (MW) per capita. Source: (59,60).

**Fig. 9:**
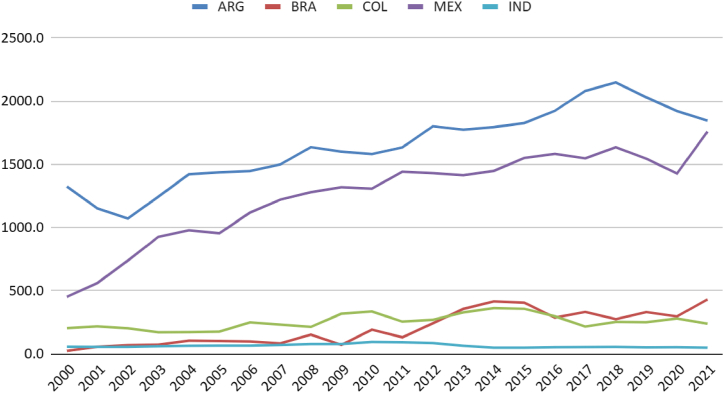
Natural gas generation (GWh) per million residents. Source: (59,60).

It is observed that Argentina and Mexico have higher per capita natural gas usage for electricity generation. The population growth and Indian demand have shown a more significant increase than the thermoelectric sector, leading to negative trends in generation and power per capita indicators (Fig. 8, Fig. 9).

Another consumption aspect is the residential, commercial, and industrial consumption of gas, excluding its use for electricity generation. Currently, 24 LDCs (Local Distribution Companies) operate in the country, serving 22 Member States. The States of Rio de Janeiro (RJ) and São Paulo (SP) are the only ones that have more than one LDC and are also those where there is (i) the highest average consumption of natural gas, (ii) the most significant network installed (45; Costa et al., 2020).

Thermoelectric and industrial consumers have a more significant share of the natural gas market. The Association of Large Industrial Energy Consumers and Free Consumers (ABRACE) represents the interests of this type of agent, representing about 42 % of the country's industrial natural gas consumption ([67]), being a proactive entity in sectoral discussions from the consumption perspective.

**Fig. 10:**
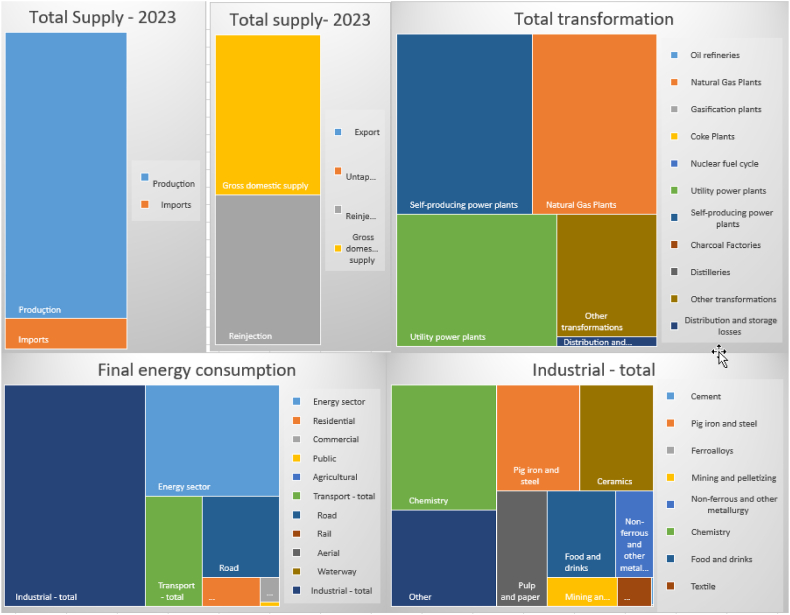
National energy balance - consolidated – 2023.

Source: (59)

In addition to the prevalence of thermoelectric and industrial consumption, it is also important to highlight that most consumers are in the country's southeast region, where a relevant part of the industry is located (Table 5 and Fig. 10). Based on 2023 data, the distribution of natural gas consumption in Brazil shows significant diversification across production, imports, and various economic sectors. Brazil internally produces 90.52 % of its total natural gas supply, while approximately 9.49 % is imported to meet national demand. A substantial portion of the natural gas, about 47.39 %, is reinjected, and 50.32 % is allocated for gross domestic supply. Within energy transformations, 24.01 % of the total supply is utilized, with notable contributions from natural gas plants (6.69 %), utility power plants (6.33 %), and self-producing power plants (7.33 %). Additionally, around 0.30 % of the gas is lost during distribution and storage. For final consumption, 26.04 % of the total supply is dedicated to this purpose, with final energy consumption accounting for 24.98 %, distributed among the energy sector (6.83 %), residential (0.77 %), commercial (0.22 %), and public sectors (0.04 %). The transport sector, responsible for 2.87 %, uses all its gas for road transportation. The industrial sector, one of the largest consumers, absorbs 14.25 % of the total, encompassing various industries such as cement, pig iron and steel, mining, chemicals, food and beverages, textiles, pulp and paper, and ceramics.

**Table 5:** National energy balance - consolidated - 2023 - 10³ Toe primary energy sources.

Account	Natural Gas	Percentage (%)
Production	54.281	90.52 %
Imports	5.692	9.49 %
Inventory variation	0	0.00 %
**Total Supply**	**59.974**	**100 %**
Export	0	0.00 %
Untapped	1.375	2.29 %
Reinjection	28.418	47.39 %
Gross domestic supply	30.181	50.32 %
Total transformation	14.403	24.01 %
Oil refineries	0	0.00 %
Natural Gas Plants	4.014	6.69 %
Gasification plants	0	0.00 %
Coke Plants	0	0.00 %
Nuclear fuel cycle	0	0.00 %
Utility power plants	3.799	6.33 %
Self-producing power plants	4.398	7.33 %
Charcoal Factories	0	0.00 %
Distilleries	0	0.00 %
Other transformations	2.192	3.66 %
Distribution and storage losses	178	0.30 %
Final consumption	15.615	26.04 %
Non-energy final consumption	641	1.07 %
Final energy consumption	14.974	24.98 %
Energy sector	4.095	6.83 %
Residential	461	0.77 %
Commercial	130	0.22 %
Public	22	0.04 %
Agricultural	0	0.00 %
Transport - total	1.722	2.87 %
Road	1.722	2.87 %
Rail	0	0.00 %
Aerial	0	0.00 %
Waterway	0	0.00 %
Industrial - total	8.545	14.25 %
Cement	4	0.01 %
Pig iron and steel	1.306	2.18 %
Ferroalloys	0	0.00 %
Mining and pelletizing	300	0.50 %
Non-ferrous and other metallurgy	473	0.79 %
Chemistry	1.938	3.23 %
Food and drinks	871	1.45 %
Textile	148	0.25 %
Pulp and paper	876	1.46 %
Ceramics	1.143	1.91 %
Other	1.486	2.48 %
Unidentified consumption	0	0.00 %
Correction	15	0.03 %

**Fig. 11:**
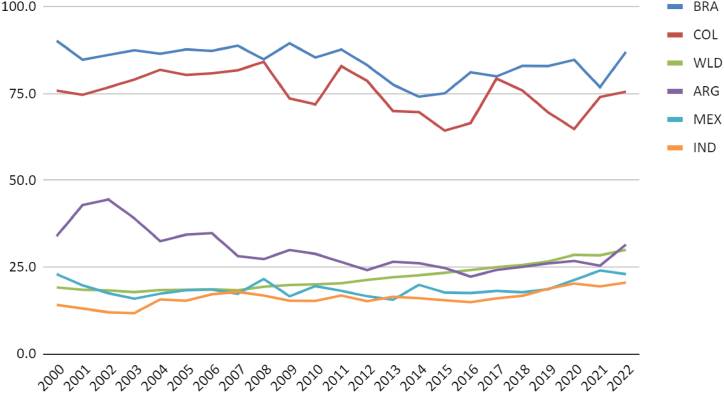
Renewables (% electricity). Source: (59,60).WLD is the world average.

Several sectors in Brazil currently use relatively small amounts of natural gas. The residential sector consumes only 0.77 % of the total supply, while the commercial sector accounts for just 0.22 %. The limited use of natural gas in Brazil's residential and commercial sectors can be attributed to the need for an improved and more competitive urban gas distribution network. With a focused program to enhance these networks, gas usage in urban areas, particularly within cities with one million populations, could grow significantly. According to recent data, Brazil has 15 municipalities with populations over one million, of which 13 are state capitals. These cities collectively house 42.7 million people, representing 20.1 % of the country's population. In the industrial sector, some specific sub-sectors demonstrate shallow consumption: the cement industry uses a mere 0.01 %, and the ferroalloy industry shows no recorded consumption. Other smaller industrial sub-sectors, like textiles, show an insignificant use at 0.25 %. These insights indicate potential opportunities to expand natural gas usage in these sectors, contributing to a more comprehensive utilization of natural gas resources in Brazil. Policies to replace coke, coal, and diesel in thermal power plants could also increase the demand for natural gas connected to the grid or LNG.

Thus, distributors located in the Southeast are responsible for serving most of the Brazilian demand, both from the volume consumed and the number of customers (Abegas, 2020).

A fundamental issue that affects large consumers and LDCs is the regulation of free consumers, self-importers, and self-producers at the state level. When these clients are regulated, the agent can choose its gas supplier, increasing their respective bargaining power and competitiveness. Some States have already edited rules defining the minimum consumption of natural gas so that an agent is considered a free consumer according to the maturity stage of its market (Costa et al., 2022) (Table 6).

**Table 6:** – Legislation defining the minimum consumption of natural gas.

State	Legislation	Minimum consumption (m3/day)
Amapá	Law 2656/2022	10.000
Amazonas	Law 5420/2021	10.000
Bahia	AGERBA Resolution 14/2021	10.000
Ceará	Law 17.897/2022	10.000
Espírito Santo	ARSP Resolution 046/2021	10.000
Maranhão	Law 11662/2022, and Law 9.102/2009	100.000
Mato Grosso	Law 7.939/2003 and Decree 1.760/2003	1.000.000
Mato Grosso do Sul	AGEPAN Order 103/2013	150.000 (industry), 500.000 (power) 1.000.000 (others)
Minas Gerais	SEDE Resolution 32/2021	5.000
Pará	Decree 1771/2017	500.000
Paraíba	Law 12.142/2021	50.000
Paraná	Law 247/2022	100.000 (power); for other consumers, the minimum is 10.000 m³/d (since May 2022)
Pernambuco	Law 17.641/2022	10.000 to 50.000
Piauí	Law 7.686/2021	10.000
Rio de Janeiro	AGENERSA Order 4068/2020 and 4142/2020	10.000
Rio Grande do Norte	Law 11.190/2022	5.000
Rio Grande do Sul	AGERGS Resolution 68/2023	No minimum consumption exists, but the consumer cannot be in the commercial or residential categories.
São Paulo	ARSESP Order 1.061/2020	No minimum consumption exists; however, the consumer cannot be in the commercial or residential categories as disciplined in the concession agreement.
Santa Catarina	ARESC Resolution 136/2019	10.000
Sergipe	AGRESE Resolution 19/2022	10.000

Table 6 is a photograph of the free consumer regulatory status in 2021 and shows that not all Brazilian States have regulations on free consumers, self-producers, and self-importers, inhibiting the development of an open natural gas market (45). Given the above data, the industrial and thermoelectric markets may continue responsible for natural gas consumption. These consumption segments are predominantly installed in the southeast region, where there is already a vast pipeline distribution network.

Between 2019 and 2021, two contracts for the transportation of natural gas from TBG (TCQ Brazil Contract and TCX Brazil Contract) ended, leading to the need for new natural gas contracts of around 24 million m³/day ([68]). As a result, LDC began proactively seeking new ways to meet their demand, diversifying their portfolio, including liquefied natural gas (LNG), and consequently increasing their bargaining power. Some geographically close LDCs have launched coordinated public calls for the contracting of natural gas, such as the south-central region ([69]).

The possibility of buying gas through a public call means new momentum for LDCs' bargaining power, as they can now reach new suppliers other than Petrobras. In previous years, some LDCs bought gas from different suppliers using public calls to choose better supply conditions ([70]), including Law 14,182/2022 provisions, as shown in Box 1. Similarly, about twelve new gas suppliers have encouraged industry players to migrate to the free market, looking for better supply conditions ([71]). Petrobras’s gradual decline as a supplier leads to more bargaining power for LDCs and industry players.

Box 1 **Law 14,182/2022 and the expansion of piped gas networks**

Another relevant change in the Brazilian gas market was the approval of Law 14182 in 2022 (Eletrobras' privatisation law), which established mandatory contracting of 8 GW of gas-fired capacity by 2030 in all regions except the south. Eletrobras, Latin America's largest utility, was formerly a state-owned power company and underwent a public share offering. The new law makes contracting gas-fired power plants mandatory in "Federation units that do not have natural gas supply in their capital" (art. 1, §1). This change is relevant because it may lead to the development of thermopower plants that could serve as anchors for developing local piped gas networks, bringing natural gas to locations not yet served, especially in the country's northern region. The industry and the final consumer are expected to enjoy the benefits of natural gas ([72]). The first power generation tender to hire the gas-fired power plants resulting from this law was scheduled for September 2022. However, it was postponed ([73]).

### 5.3 Threat of new entrants

The presence of several gas producers, shippers, distributors, and traders can provide better purchase conditions to serve their customers in terms of price and general conditions of the service provided, including greater security in supply. This change generates alternatives for the front of the chain, benefiting large consumers. The Brazilian gas industry has historically presented vertical integration as its main characteristic since practically a single company - Petrobras, controlled production, the main transport pipelines, offering gas and transmission services in an integrated way. The more limited access to channels, wholesale and retail, and the greater the control over these channels, the more difficult entry into the industry will be (57).

From a production (upstream) perspective, the opening of the market due to the end of Petrobras' monopoly in 1997 put down the legal barrier that was in place, preventing new players from entering the market. Since the country observed several different companies populating the market both in the offshore and onshore environments, a tendance that has been boosted by Petrobras’ divestment policy over the last years, indicating a reduction in consumer dependence on the company ([72,74]).

Regarding transport pipelines, Brazil's gas industry is still very restricted. Although Petrobras has sold TAG and NTS gas pipeline networks, for instance, as part of the agreement signed with CADE in 2019, the company maintained contracts for using the infrastructure for the following decades, with renewal clauses (69). As a result, even though public calls made by the new gas pipeline owners made room for gas transport from other companies, the transport availability was relatively small. It did not implicate changes in the price structure defined by the State-owned company (40).

Despite the challenging scenario, it is expected that, after the approval of the New Gas Law, non-discriminatory access to the gas transport pipelines will effectively happen (69).

The gas distribution segment was also part of the Petrobras-CADE agreement. Petrobras committed to selling Gaspetro, a former Petrobras subsidiary through which the state-owned company had shares in several different LDCs. The divestment initiative was expected to allow new players to enter the market. The divestment procedure was completed in July 2022, and Compass and Mitsui Gas e Energia assumed rebranding the company to Commit (54; 55; 16).

As Porter (22 points out, companies from other markets diversifying through acquisition or entry into a particular segment often employ substantial resources to cause changes, such as differentiation mechanisms, about initial participants. This change, whether in better conditions in terms of the quantity offered, prices, guarantees and forms of contracts (take-or-pay, ship-or-pay), is substantially significant and of crucial interest for large consumers and the competitive development of the gas industry (69; 52). Although very recent, the changes in the distribution segment in Brazil have already shown Porter's (22) prediction are valid: LDCs that were somehow influenced by Petrobras' divestment are investing and expanding their grids and searching for new gas sources, including biomethane ([75]).

In the transmission and distribution/sales segment, despite the New Gas Law being approved in 2021, we still cannot perceive its effects, such as promoting new investments and attracting new companies. (52). Several regulations are still needed and waiting for the transition phase to pass so that access to transport pipelines is accessible. The use of greater competition in production, transmission, and distribution, as well as new alternatives in supply conditions, are the benefits expected by large consumers (industries).

### 5.4 Threat of substitution

In the Brazilian market, a large portion of the natural gas potential market is currently supplied by other petroleum derivatives, mainly fuel oil and LPG, both for industrial and thermoelectric markets, the main ones responsible for gas consumption in the country.

Natural gas faces the most considerable number of competitors in the industrial segment. It competes with petroleum products such as fuel oil, diesel oil, LPG, biomass (firewood and sugar cane pulp), hydrogen and electricity power ([76]). In industries that primarily use fuel oil and LPG, in addition to the required gas supply availability, the switch to natural gas depends on technical, economic, logistical, and environmental feasibilities, among other topics that can appear to a specific consumer situation (39).

Between 1999 and the construction of the Gasbol pipeline and the first decade of the 2000s, a series of initiatives were adopted to stimulate the use of natural gas in the country. The low-price policies stimulated many industrial sectors to adapt their production plants to use natural gas instead of fuel oil, which is more expensive and aggressive to the environment. Industries such as glass and ceramics, for example, could enjoy the benefits of using natural gas in their manufacturing processes since this energy source is cleaner and generates less waste. Additionally, product quality has increased, raising their product competitiveness in the market not only due to the cost competitiveness but also due to higher product quality. Another example is the textile and leather industries, which also could get advantages from access to lower production costs and higher product quality ([77]).

However, at that time, a perspective of natural gas scarcity shortly increased natural gas prices. In the following years, it significantly impacted the industrial sector, reducing consumers' confidence and fomented investments in dual-fuel equipment (natural gas and another oil derivative). As a result, the demand for natural gas in the industry became more vulnerable to the natural gas market conditions. In the following years, natural gas gradually recovered its competitiveness against fuel oil ([78]). The fuel substitution continues to be limited to the regions where gas supply alternatives (mainly through gas pipelines and, more recently, via small-scale LNG or CNG projects) can be found. Brazil counts on 27 thousand kilometres of gas pipelines. According to Fórum do Gás, this is equivalent to 2 % of the US gas pipeline network, less than the total length of gas pipes in Argentina and Mexico and about the same as what can be found in Indonesia and Hungary.

As previously described, the thermoelectric market is also a large natural gas consumer. Even though gas-fired electricity generation is not a direct substitute for natural gas, existing contracts prioritise supply to thermal power plants, implying fewer options and less flexible conditions for consumers ([74]).

On the other hand, due to the characteristics of the Brazilian electric power system, gas-fired power generation is the most suitable option for power generation and consumption. From the perspective of fuel for power generation, renewable power, mainly solar and wind, might appear as a substitute for using natural gas for power generation. These power sources show a competitive cost worldwide once the fuel is free of charge ([79]). It puts pressure on the government to reduce the price of power in the future. This situation may only change once energy storage costs remain high.

Considering the threat of substitution, Brazilian natural gas demand in the industry is susceptible to natural gas market conditions (40). It is necessary to ensure a competitive price and continuous supply for a more solid consumption. Electric power generation is, and will most likely continue to be, strongly demanded until a competitive energy storage technology reaches the breakeven cost. Finally, from the final consumer perspective, natural gas is highly competitive, and it is challenging to lose the market when the final consumer has access. The competitiveness of the final consumer market should be explored more through the expansion of natural gas pipelines and deregulation. It will achieve an even more competitive price to reach infrastructure capillarity ([78]).

Hydrogen can also be a vital substitute for natural gas since it is being used in several applications that currently belong to natural gas and can be transported worldwide. The likely target sectors in Brazil are the cement, mobility, and fertiliser industries ([80]). In addition, hydrogen can use the current natural gas distribution structure, which increases the substitution that threatens power ([81]). Due to Brazil's extensive renewable energy availability, green hydrogen will become a solid threat to natural gas, especially considering the energy transition picture ([82]). Finally, other studies suggest Brazil can achieve a crucial global position in the hydrogen market globally ([83]).

**Fig. 12:**
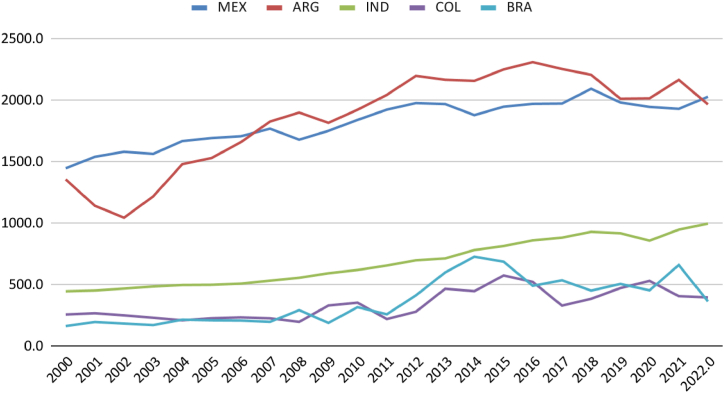
Electricity from fossil fuels kWh per capita. Source: (59,60).

**Fig. 13:**
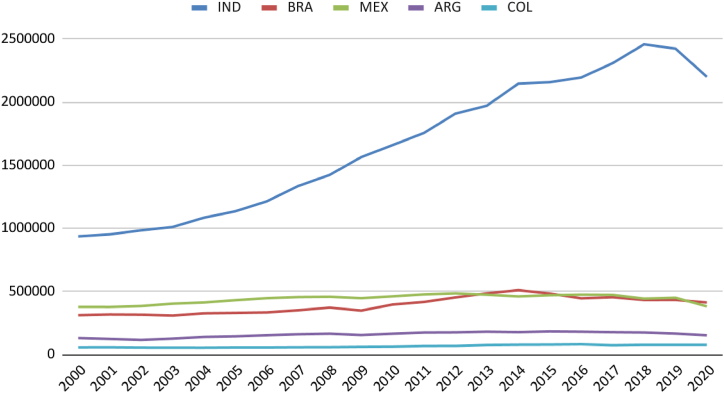
CO2 emissions (kt). Source: (59,60).

**Fig. 14:**
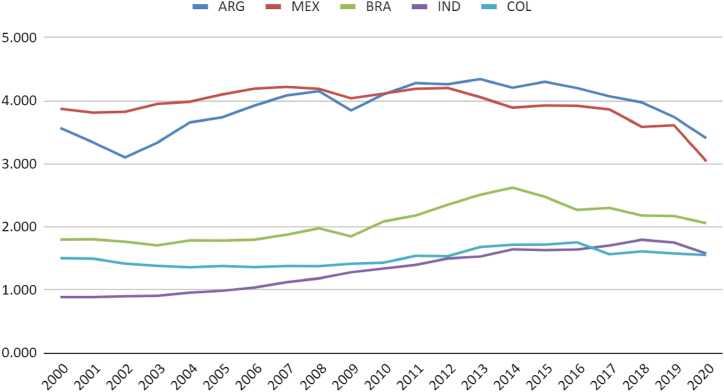
CO2 emissions (kt) per 1000 residents. Source: (59,60).

**Fig. 15:**
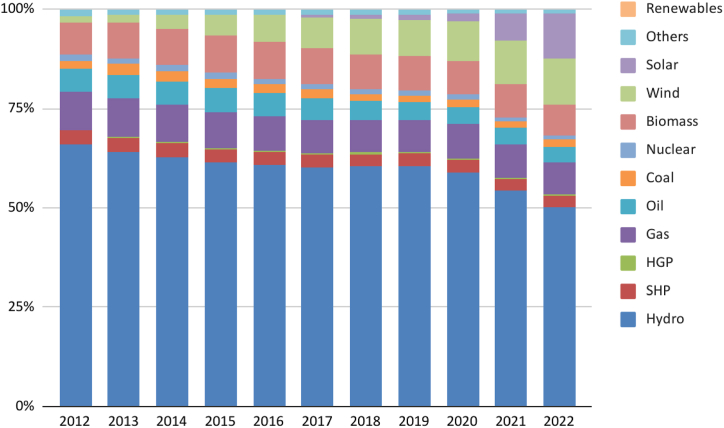
Brazil’s electricity installed capacity (MW) Sources: ([84]). SHP (Small Hydroelectric Plants) and HGP (Hydroelectric Generating Plants) represent two categories of hydroelectric facilities distinguished primarily by their energy generation capacity. Small Hydroelectric Plants (SHP) encompass hydroelectric installations with an installed capacity exceeding 1 MW and up to 30 MW, typically featuring reservoir areas of up to 3 km^2^. On the other hand, Hydroelectric Generating Plants (HGP) refer to smaller hydroelectric facilities with an installed capacity of up to 1 MW. These plants are commonly employed to cater to the energy needs of smaller communities or enterprises, boasting an even lesser environmental footprint compared to PCHs.

Another significant competitor to thermal power plants is electricity production from renewable sources (15, 16; 17). Over the last four years, wind and solar sources have surpassed gas-fired thermal power plants in the national electrical grid, providing greater diversity alongside hydroelectric and biomass sources. Another advantage of these sources over gas is the positive impact of renewable sources on the electricity grid and carbon emission reduction. In this aspect, comparing Brazil with the selected countries demonstrates that Brazil and Colombia are well above the global average, with approximately 90 % of the electricity grid based on renewables (Fig. 11). Considering the long-term data of 20 years, all countries show a positive trend for per capita growth of fossil fuels in the electricity grid. However, a negative trend is observed in Latin American countries considering the medium term of the last five years of the historical series (17). The more intensive use of fossil fuels is observed in Mexico and Argentina. India shows an almost constant growth over time, approaching the per capita consumption of the two leading selected countries (Fig. 12). Latin American countries show, in the first fifteen years of the historical series, a growth in total emissions and per capita emissions (Fig. 13, Fig. 14). The last quartile of the historical series already shows a downward trend in emissions. An exception is identified in India's exponential growth in emissions due to the urbanisation process and the increase in GDP PPP per capita. However, population growth still outpaces emission growth, resulting in a decline in per capita rates. Although the gas industry is less polluting than the two other fossil fuels, it contributes to the decrease in the performance of these countries regarding emissions. However, the energy security of these countries requires the maintenance of thermal power systems, as is the case with Brazil and Colombia, with a significant participation of hydroelectric power (Fig. 15).

### 5.5 Degree of rivalry

Porter (22) states that high fixed costs, asset specificity and exit barriers indicate intense market competition. In addition, factors that promote a more competitive environment include company commitment and goals that go beyond profit maximisation. The extent of internal competition in a company, which depends on the competition of agents and the restrictions established, is a factor in its competitiveness (regulatory environment). Porter (22) also states that it is more challenging to form monopolies, oligopolies, and collaborations the more business, overtly or covertly, there is in a given area.

Historically, Petrobras is a monopolist in the Brazilian gas market, operating vertically and controlling most of the market’s infrastructure. The company has limited competition even in competitive market segments or indirect influence or control over less competitive segments of the chain (transportation and distribution, production, and commercialisation. The vertical integration between competitive and non-competitive links prevented competition from evolving.

A robust regulatory framework would be required to protect the market and promote competition, prohibiting companies that dominate non-competitive (regulated) segments from abusing their position to engage in activities that harm the competitive stages of the chain ([85]). Alternatively, changing the picture would require dividing the corporation into distinct or smaller sectors.

As described before, market opening, recent divestment decisions, and regulatory developments pointed towards it, changing the scenario and promoting a more competitive landscape.

The upstream sector, which includes exploration, production, and transportation, faces a relatively high degree of rivalry due to multiple players in the market and the possibility of new entrants since the market opening 1997. The presence of several different companies in the market, both in the offshore and onshore environments, led to a relatively high degree of rivalry among them, bringing competition to the bid rounds, more competitive pricing, and a more efficient allocation of resources ([72,86]).

The midstream segment, which includes processing, storage, and transportation of natural gas, faces a lower rivalry due to the high capital requirements and the dominance of a few significant players – even considering Petrobras' divestments. Hence, competition is limited at this point of the value chain, and higher entry barriers can be found.

Last, the downstream segment is characterised by natural monopolies with no direct competitors ([87]). Each local distribution company has a unique situation that influences its investment decisions. Since LDCs operate under concession areas, they do not compete with each other. However, they compete in different ways with a substitute or alternative energy sources (e.g. LPG, diesel and fuel oil), sometimes with intense rivalry when different agents compete for customer "preference".

In the natural gas market, an extensive network where access decisions can be made by a dominant market agent, using policies to diversify the referred network is natural thinking. The policy improvement could be achieved under laws, regulation or the use of a Competition Law, in some cases, the latter being indicated when there is an unjustified refusal of access to an "essential facility" of the market or in the absence of an unbundling policy (43; 74).

The tariffs imposed by natural gas distributors on their customers are the first sensitive competitive variable subject to intensive regulation and, therefore, excluded from the control or influence of Local Distribution Companies. Those concessionaires do not apply prices but public tariffs, which are determined by the respective state regulatory body based on a detailed methodology in the concession contracts and per transparency, universality, and economic-financial balance criteria ([88]).

Quantities delivered are the second sensitive competition variable away from the control or influence of LDCs. As a public service, piped gas obeys the principles of generality (the service must be the same for everyone) and continuity (the service must be successive and cannot suffer interruption of continuity). In addition, the concession contracts signed by the concessionaires with the state government usually include specific targets for investment and expansion of the distribution network system for a reasonable and cost-effective approach to the service. (39).

In addition, mainly because of market development, more and more traders have been able to buy natural gas directly from producers, importers and other traders and resell it to LDCs or end consumers. Distribution companies sell the molecules to end customers in the so-called captive or regulated market. Suppliers (producers and importers of natural gas and LNG) trade natural gas with each other and other traders, distribution companies and end consumers ([89]; 48). Production, importation, and especially commercialisation are portions of the value chain that have the potential for competition, but transmission and distribution present the characteristics of natural monopolies.

## 6 Conclusion and policy recommendations

The data analysis shows that Brazil's gas industry has significantly transformed over the past twenty years. Regarding generation and absolute capacity, it has transitioned from one of the smallest to one of the most productive among the selected countries. Consequently, despite the growth in electricity generation and oil production, especially following oil reserve exploration, the share of fossil fuels in the electricity grid has remained the same. Upstream production gradually shifted away from the state monopoly of the 1990s to approximately 70 % by the end of the analysed period. Petrobras still dominate gas generation and hold a considerable stake in thermal power generation. The increase in gas production has ensured that Brazil's electricity grid remains predominantly reliant on renewable energy sources, maintaining levels close to 90 %, and has prevented the growth of more polluting sources such as coal, oil, and nuclear power. Thermal power plants ensure energy security during drought and reduce hydroelectric production.

The country's gas production has gradually replaced some imported gas, although nearly half of the produced gas is reinjected into mature wells to maintain oil productivity. Furthermore, the inadequate midstream infrastructure for gas distribution across the country due to low penetration in the interior and significant cities remains a challenge. Despite initiatives to liberalise the upstream, midstream, and downstream sectors, Brazil stands out as the country that utilises the most public resources for energy investment among the researched nations. This investment has contributed to the increase in gas reserves and production. However, the commercialisation of the produced gas has yet to be adequately developed, even when compared to the selected countries.

Internal regional consumption disparities are not solely due to economic inequality but also stem from the lack of access to gas pipelines in the interior, urban peripheral areas, and less industrialised states in the north and northeast. This dependency on LNG imports persists despite the potential and production capabilities of the upstream sector. Therefore, public policies must be implemented across various dimensions to address these challenges.

Regulatory Framework Enhancement.

1. Ensure adequate technical and financial resources to implement the regulatory agenda proposed under the "Novo Mercado do Gás" program by the National Agency of Petroleum, Natural Gas and Biofuels (ANP).
2. Enact clear and transparent criteria for free customers at the state level as part of the New Gas Law (Law 14.143/21) to foster competition and reduce supplier bargaining power while enhancing customer bargaining power.
3. Achieve harmony and integration between federal and State regulatory regimes and agencies to facilitate market opening and address discrepancies hindering market development.

Infrastructure Development.

1. Address structural challenges in the vertically integrated chain, including expanding infrastructure and reducing Petrobras' supplier bargaining power. A regulatory measure to reduce the reinjection of natural gas is imperative to increase the availability of natural gas from the pre-salt reserves. This factor would impact the prices of natural gas available in the domestic and external markets.
2. Facilitate gas release processes to stimulate competition, particularly in the upstream sector, where Petrobras still holds a significant share of gas production.

Market Competition Promotion.

1. Implement measures to stimulate competition, such as facilitating gas release processes and ensuring competitive pricing and continuous supply to maintain solid natural gas consumption.
2. Address discrepancies between federal and State regulations to encourage customer migration to the free market and enhance market development.

Energy Transition Promotion.

1. Promote natural gas as a transition fuel towards sustainability, considering its significant role in Brazil's energy matrix alongside renewable sources.
2. Attract investments in the natural gas sector by highlighting its contribution to energy security and its potential to reduce greenhouse gas emissions.
3. Encourage research and development initiatives to explore the potential of natural gas in the energy transition and identify opportunities for technological advancements.

By addressing these critical areas through targeted policy measures, Brazil can further enhance the competitiveness and profitability of its natural gas industry while contributing to its energy security and sustainability goals.

This study identifies areas for future research and development. One avenue involves developing a computational mathematical model to examine how fluctuations in international oil prices and interest rates affect energy investments across the selected countries. Furthermore, there is potential for creating a computational engine capable of quantitatively assessing the various internal factors influencing these indicators. Such tools could offer valuable insights into the long-term impacts of public policies on energy markets.

## CRediT authorship contribution statement

**Hirdan Katarina de Medeiros Costa:** Writing – review & editing, Validation, Supervision, Project administration, Methodology, Investigation, Funding acquisition, Formal analysis, Data curation, Conceptualization. **Rafael Sacco:** Writing – review & editing, Writing – original draft, Investigation, Data curation. **Clarissa Emanuela Leão Lima:** Writing – review & editing, Writing – original draft, Investigation, Formal analysis. **Rodrigo Botão:** Writing – review & editing, Writing – original draft, Investigation, Formal analysis, Conceptualization. **Ciro Galvão:** Writing – review & editing, Writing – original draft, Visualization, Investigation. **Gabriela Pantoja Passos:** Writing – review & editing, Writing – original draft, Methodology, Investigation, Formal analysis. **Thiago Brito:** Writing – original draft, Investigation, Formal analysis, Conceptualization. **Giancarlo Ciola:** Writing – review & editing, Writing – original draft, Investigation. **Marcos Eduardo Melo dos Santos:** Writing – review & editing, Writing – original draft, Methodology, Investigation, Funding acquisition, Formal analysis, Conceptualization. **Jewellord Nem Singh:** Validation, Supervision, Methodology, Formal analysis, Conceptualization. **Edmilson Moutinho dos Santos:** Writing – review & editing, Writing – original draft, Supervision, Project administration, Methodology, Investigation, Funding acquisition, Formal analysis, Conceptualization.

## Data and code availability statement

Corresponding data is available on request.

## Funding

This research was funded by 10.13039/501100006487ANP/10.13039/501100004809FINEP/FUSP/10.13039/501100005639USP, grant number 0443/19, and 10.13039/100017586RCGI/10.13039/501100001807FAPESP, grant number 2020/15230-5.

## Declaration of competing interest

The authors declare the following financial interests/personal relationships which may be considered as potential competing interests: Hirdan Costa reports financial support was provided by Agência Nacional de Petróleo, Gás Natural e Biocombustíveis (ANP). Rafael Luís Sacco reports financial support was provided by Agência Nacional de Petróleo, Gás Natural e Biocombustíveis (ANP). Rodrigo Botão reports financial support was provided by Agência Nacional de Petróleo, Gás Natural e Biocombustíveis (ANP). Gabriela Passos reports financial support was provided by Agência Nacional de Petróleo, Gás Natural e Biocombustíveis (ANP). Thiago Luis Felipe Brito reports financial support was provided by Agência Nacional de Petróleo, Gás Natural e Biocombustíveis (ANP). Murilo Tadeu Werneck Fagá reports financial support was provided by Agência Nacional de Petróleo, Gás Natural e Biocombustíveis (ANP). Edmilson Moutinho dos Santos reports financial support was provided by Agência Nacional de Petróleo, Gás Natural e Biocombustíveis (ANP) and by Research Centre for Greenhouse Gas Innovation - 10.13039/100017586RCGI (RCGI). Marcos Eduardo Melo dos Santos reports financial support was provided by 10.13039/100019577Virtual University of São Paulo and Erasmus University of Rotterdam.
